# Correlates of meeting the combined and independent aerobic and strength exercise guidelines in hematologic cancer survivors

**DOI:** 10.1186/s12966-017-0498-7

**Published:** 2017-03-28

**Authors:** James R. Vallerand, Ryan E. Rhodes, Gordon J. Walker, Kerry S. Courneya

**Affiliations:** 1 Faculty of Physical Education and Recreation, University of Alberta, Edmonton, AB Canada; 2 School of Exercise Science, Physical & Health Education, University of Victoria, Victoria, BC Canada

**Keywords:** Physical activity, Multi-process action control framework, M-PAC, Intention-behavior gap

## Abstract

**Background:**

Most previous research on the correlates of physical activity has examined the aerobic or strength exercise guidelines separately. Such an approach does not allow an examination of the correlates of meeting the combined guidelines versus a single guideline, or one guideline versus the other. Here, we report the prevalence and correlates of meeting the combined and independent exercise guidelines in hematologic cancer survivors (HCS).

**Methods:**

In a population-based, cross-sectional survey of 606 HCS from Alberta, Canada using a mailed questionnaire, we obtained separate assessments of aerobic and strength exercise behaviors, as well as separate assessments for motivations, regulations, and reflective processes using the multi-process action control framework (M-PAC).

**Results:**

Overall, 22% of HCS met the combined exercise guideline, 22% met aerobic-only, 10% met strength-only, and 46% met neither exercise guideline. HCS were more likely to meet the combined guideline over the aerobic-only guideline if they had no children living at home, and over both the aerobic and strength-only guidelines if they had completed university. As hypothesized, those meeting the combined guideline also had a more favorable *strength-specific* M-PAC profile (i.e., motivations, regulations, and reflective processes) than those meeting the aerobic-only guideline, and a more favorable *aerobic-specific* M-PAC profile than those meeting the strength-only guideline. Interestingly and unexpectedly, HCS meeting the combined guidelines also reported significantly greater *aerobic-specific* perceived control, planning, and obligation/regret than those meeting the aerobic-only guideline, and greater *strength-specific* perceived control, planning, and obligation/regret than those meeting the strength-only guideline.

**Conclusions:**

Few HCS are meeting the combined exercise guidelines. M-PAC based variables are strong correlates of meeting the combined guidelines compared to aerobic or strength only guidelines. Strategies to help HCS meet the combined guidelines may need to promote more favorable behavioral regulations and reflective processes for both types of exercise rather than just the type of exercise in which HCS are deficient.

**Electronic supplementary material:**

The online version of this article (doi:10.1186/s12966-017-0498-7) contains supplementary material, which is available to authorized users.

## Background

To improve cancer survivors’ physical functioning and quality of life [1, 2], the American College of Sports Medicine (ACSM) recommends that survivors accumulate a weekly total of at least 150 min of moderate-to-vigorous aerobic exercise (aerobic guideline), and two weekly sessions of strength training that target the major muscle groups (strength guideline) [3]. Complying with this combined guideline serves as the optimal scenario for cancer survivors, as it affords them the unique benefits of both aerobic and strength exercise guidelines (e.g., cardiovascular health, body composition, physical functioning). Thus, understanding the determinants of the combined guideline is critical to the health of cancer survivors.

Previous research on the correlates of exercise has generally reported the correlates of “physical activity,” with more recent efforts detailing the correlates separately for aerobic and strength exercise [4, 5]. Crawford and colleagues have recently argued that the correlates of meeting the combined guideline may be different than a single guideline, and different strategies may be needed to motivate individuals to perform the combined guideline if they already meet one guideline or neither [6]. To explore these concepts, researchers need to examine the correlates of aerobic and strength exercise simultaneously. In an important first step, Crawford and colleagues followed this approach using a dataset of demographic and clinical variables, but these variables did not meaningfully distinguish between survivors meeting the combined guideline versus either single guideline [6]; prompting them to suggest that exercise-specific social cognitive variables may better differentiate these groups.

The purpose of the present study is to examine the correlates of meeting the combined and independent exercise guidelines in a population-based sample of hematologic cancer survivors (HCS). In our recent population-based survey of over 600 Albertan HCS, we examined their aerobic and strength exercise participation and motivations [7, 8], and focused our examination around a theoretical framework (the multi-process action control framework; M-PAC) which explicitly accounts for motivational (e.g., attitude, perceived control), regulatory (e.g., planning), and reflective (e.g., habit, identity) differences that characterize the gap existing between intention and behavior (known as the intention-behavior gap; I-B Gap) [9, 10]. A particular strength of our study was that we assessed M-PAC based variables separately for both aerobic and strength exercise. In line with previous research, however, we reported the prevalence and correlates of aerobic and strength exercise separately. We now believe that a more informative approach would be to consider the correlates of both guidelines simultaneously.

We organized our hypotheses into themes: “traditional” and “novel.” Our traditional hypotheses involved comparing demographic, cancer, and M-PAC based variables (i.e., motivational processes, behavioral regulations, and reflective processes) of HCS meeting the three exercise guidelines (combined, aerobic-only, and strength-only) versus neither guideline. Our novel hypotheses involved comparing these same variables between each of the three exercise guidelines. Our *traditional* hypotheses were that key demographic and cancer-specific variables would differentiate HCS meeting either of the three exercise guidelines versus those meeting neither. Regarding the M-PAC based variables, we made the *traditional* hypotheses that compared to HCS meeting neither guideline, those meeting the combined or aerobic guidelines would have a more favorable aerobic-specific M-PAC profile, and those meeting the combined or strength guidelines would have a more favorable strength-specific M-PAC profile. In terms of *novel* hypotheses, we hypothesized that key demographic and cancer-specific variables would also differentiate HCS meeting the combined guidelines versus those meeting the aerobic-only and strength-only guidelines, and may even distinguish those meeting aerobic-only versus strength-only. We also made the *novel* hypotheses that those meeting the combined guideline would have a more favorable strength-specific M-PAC profile versus those meeting the aerobic-only guideline, and a more favorable aerobic-specific M-PAC profile versus those meeting the strength-only guideline. Finally we expected large differences in the aerobic and strength specific M-PAC profiles of those meeting the aerobic-only versus strength-only guideline.

## Methods

The design and methods of our survey study have been detailed elsewhere [7]. Briefly, ethical approval and informed consent were obtained for all procedures performed in the study. A stratified random sample of 2100 adult HCS (700 of each leukemia, Hodgkin lymphoma, and non-Hodgkin lymphoma) was contacted by the Alberta Cancer Registry to participate in this study. Data was collected from self-report questionnaires, where participants completed surveys by hand and returned them via post.

### Measures

#### Demographic and cancer-specific variables

Demographic variables included age, sex, marital status, education, employment status, ethnicity, height, and weight. Cancer-specific variables included cancer type, previous treatments, time-since-diagnosis, current treatment status, cancer recurrence, current cancer status (existing disease versus disease-free), comorbidities, and whether participants received an exercise recommendation by one of their healthcare professionals involved in their cancer treatment.

#### Aerobic and strength exercise behavior

A modified version of the Godin Leisure Time Exercise Questionnaire (GLTEQ) was used to measure exercise behavior [11]. As the original GLTEQ did not separate aerobic and strength exercise, we included separate aerobic and strength questions. Participants were asked to first indicate the frequency and duration of any light, moderate, and vigorous aerobic exercise (i.e., exercise that improves the heart and lungs such as walking or running), they would have completed in a typical week over the past month. Participants were then asked to indicate the average frequency (days/week) and duration (minutes/session) of any moderate-to-intense strength exercise (i.e., exercise that improves muscular strength such as weight lifting, resistance bands, sit-ups, push-ups) that they performed in a typical week over the past month. Weekly moderate-to-vigorous aerobic minutes were totaled, with vigorous minutes double weighted. Exercise levels were then dichotomized according to their respective guideline (i.e., aerobic: < or ≥ 150 min; strength: < or ≥ 2 sessions per week) [3]. Based on this information, we created a composite exercise guideline variable which categorized each HCS as meeting one of the following guidelines: neither, aerobic-only, strength-only, or combined.

#### Aerobic and strength exercise intention

In line with the M-PAC, the decision to form an exercise intention was measured using two separate dichotomous items, one reflecting an intention to meet the aerobic guideline and the other to meet the strength guideline (i.e., “Do you intend to do regular aerobic/strength exercise over the next month? Yes/No”) [12]. The term “regular exercise” was defined to reflect the levels of exercise that would constitute either the aerobic or strength guideline respectively (i.e., aerobic: ≥ 150 weekly minutes; strength: ≥ 2 weekly sessions). Again, we used this information to create another composite variable which categorized each HCS as either having an intention to meet the following guideline: neither, aerobic, strength, or combined.

#### Motivational processes

All of the following questionnaire items were first asked in relation to aerobic exercise and then repeated for strength exercise in a separate section of the questionnaire. Standard measures from the theory of planned behavior (TPB) assessed survivors’ exercise motivation on a 7-point bipolar Likert scale [13, 14]. Six items captured attitude (e.g., useful-useless, enjoyable-unenjoyable). Three items measured injunctive norm (e.g., “… people who are important to me will be…” encouraging-discouraging), and three items captured descriptive norm (e.g., “… people who are important to me will perform…” regular aerobic/strength exercise-no aerobic/strength exercise). Three items measured perceived control (e.g., “… regular aerobic/strength exercise over the next month would be completely up to me …” strongly agree-disagree).

#### Behavioral regulations

Five items assessed exercise plans (when, where, and what type) using a 7-point scale (i.e., no plans – detailed plans) [15].

#### Reflective processes

Two items measured anticipated regret (e.g., “If I do not engage in regular aerobic/strength exercise over the next month, I will feel regret.”) on an 11-point scale (i.e., definitely no – definitely yes) [16]. Exercise obligation and regulation over alternative activities were assessed using seven items on a 10-point scale (i.e., completely true for me - not at all true for me) [17]. Three items assessed exercise obligation (e.g., “I feel obligated to do regular aerobic/strength exercise over the next month…”) and four items measured self-regulation over alternative competing activities (e.g., “Compared to doing regular aerobic/strength exercise over the next month, there are other things I could do which would be more fun…”). Items for self-regulation over competing alternative activities were reverse scaled so that higher scores would reflect greater self-regulation over competing activities.

### Statistical analyses

Factor structures for the motivational, regulatory, and reflective variables are presented elsewhere [7, 8]. Briefly, separate exploratory factor analyses yielded identical seven factor structures for aerobic- and strength-specific M-PAC based variables (i.e., planning, obligation/regret, attitude, self-regulation over alternatives, descriptive norm, injunctive norm, and perceived control). Attitude, descriptive norm, injunctive norm, perceived control, and planning scores ranged from 1 to 7, self-regulation over alternatives ranged from 1 to 10, and obligation/regret scores ranged from 1 to 10.4 because this factor combined two anticipated regret items (measured on 1–11 scales) and 3 obligation items (measured from 1 to 10). Descriptive statistics were used to estimate the prevalence of guideline adherence and the magnitude of the I-B gap. Multivariate analyses of variances (MANOVA) and chi-square analyses were used to examine differences in demographic, cancer, and M-PAC based variables between the four levels of guideline adherence. Any demographic or cancer variables that emerged significant in chi-square analyses were entered into a multinomial logistic regression to assess post hoc differences among the four guideline groups. Post hoc pairwise comparisons were conducted to interpret significant between-group differences for the M-PAC based variables.

## Results

Participant flow through the study and characteristics are presented elsewhere [7]. Briefly, 29% of those contacted for this study returned a completed survey (606/2100). Excluding return-to-senders and deceased persons yielded a 32% response rate (606/1921). Overall, 186 (31%) participants were leukemia, 187 (31%) Hodgkin lymphoma, and 233 (38%) non-Hodgkin lymphoma survivors. Based on limited data from the cancer registry, responders did not significantly differ from non-responders on age, sex, disease stage, and time since diagnosis, but were more likely to be non-Hodgkin lymphoma survivors (*p* < .001) and to have received chemotherapy (*p* = .017).

### Prevalence of HCS intending and meeting the exercise guidelines

Table 1 reports the prevalence of intending and meeting the combined and independent exercise guidelines, as well as the I-B gap. Overall, 22% (134/606) of HCS met the combined guideline, 22% (133/606) met aerobic-only, 10% (58/606) met strength-only, and 46% (281/606) met neither guideline. In terms of exercise intention, 51% (312/606) intended to meet the combined guideline, 19% (116/606) the aerobic-only, 7% (41/606) the strength-only, and 23% (137/606) neither guideline. In terms of the I-B gap, 40% (127/312) of HCS with an intention to meet the combined guideline, 44% (51/116) with an intention to meet the aerobic-only, 37% (15/41) with an intention to meet the strength-only, and 90% (124/137) with an intention to meet neither guideline, acted in accordance with their intention.

**Table 1:** The intention-behavior relationship for meeting neither, aerobic-only, strength-only, or combined exercise guidelines

	Behavior			
Intention	Neither (*n* = 281)	Aerobic (*n* = 133)	Strength (*n* = 58)	Combined (*n* = 134)
Neither (*n* = 137)	124 (90%)	8 (6%)	4 (3%)	1 (1%)
Aerobic-only (*n* = 116)	59 (51%)	51 (44%)	2 (2%)	4 (3%)
Strength-only (*n* = 41)	24 (58%)	0 (0%)	15 (37%)	2 (5%)
Combined (*n* = 312)	74 (24%)	74 (24%)	37 (12%)	127 (40%)

### Correlates of meeting neither, aerobic, strength, or combined guidelines

Results from the chi-square analyses relating demographic and cancer variables to the four guideline categories are reported in Tables 2 and 3. Significant univariate associations emerged for age, education, employment status, number of children living at home, cancer type, cancer status, and comorbidities. When entered into a multinomial logistic regression, only the associations with age (*p* = .008), number of children living at home (*p* = .010), and education (*p* < .001) remained independently significant (*R* ^*2*^ = .17, *p* < .001). In terms of M-PAC profiles (Table 4), the MANOVA revealed significant main effects for each aerobic- and strength-specific motivational, regulatory, and reflective variable (all *p*s < .001).

**Table 2:** Demographic profile of survivors meeting neither, aerobic-only, strength-only, or combined exercise guidelines

Variable	Neither (*n* = 281)	Aerobic (*n* = 133)	Strength (*n* = 58)	Combined (*n* = 134)	*p* value
Age					<.001
< 60 years (*n* = 303)	99 (33%)	84 (28%)	28 (9%)	92 (30%)	
≥ 60 years (*n* = 303)	182 (60%)	49 (16%)	30 (10%)	42 (14%)	
Gender					.22
Female (*n* = 341)	147 (43%)	81 (24%)	31 (9%)	82 (24%)	
Male (*n* = 265)	134 (51%)	52 (20%)	27 (10%)	52 (20%)	
Body Mass Index					.14
Normal weight (*n* = 221)	96 (43%)	50 (23%)	19 (9%)	56 (25%)	
Overweight (*n* = 245)	106 (43%)	57 (23%)	25 (10%)	57 (23%)	
Obese (*n* = 140)	79 (56%)	26 (19%)	14 (10%)	21 (15%)	
Marital status					.46
Not married (*n* = 179)	80 (45%)	36 (20%)	22 (12%)	41 (23%)	
Married (*n* = 427)	201 (47%)	97 (23%)	36 (8%)	93 (22%)	
Children living at home					<.001
None (*n* = 450)	222 (49%)	82 (18%)	49 (11%)	97 (22%)	
One or more (*n* = 156)	59 (38%)	51 (33%)	9 (6%)	37 (24%)	
Education					<.001
University not completed (*n* = 295)	159 (54%)	60 (20%)	36 (12%)	40 (14%)	
Completed university (*n* = 311)	122 (39%)	73 (24%)	22 (7%)	94 (30%)	
Employment status					<.001
Not retired (*n* = 375)	144 (38%)	91 (24%)	35 (9%)	105 (28%)	
Retired (*n* = 231)	137 (59%)	42 (18%)	23 (10%)	29 (13%)	
Ethnicity					.14
White (*n* = 562)	267 (48%)	123 (22%)	53 (9%)	119 (21%)	
Other (*n* = 44)	14 (32%)	10 (23%)	5 (11%)	15 (34%)

**Table 3:** Cancer-specific profile of survivors meeting neither, aerobic-only, strength-only, or combined exercise guidelines

Variable	Neither (*n* = 281)	Aerobic (*n* = 133)	Strength (*n* = 58)	Combined (*n* = 134)	*p* value
Cancer type					.008
Leukemia (*n* = 186)	96 (52%)	37 (20%)	17 (9%)	36 (19%)	
Hodgkin lymphoma (*n* = 187)	64 (34%)	47 (25%)	21 (11%)	55 (29%)	
non-Hodgkin lymphoma (*n* = 233)	121 (52%)	49 (21%)	20 (9%)	43 (19%)	
Time since diagnosis					.11
< 2 years (*n* = 116)	53 (46%)	19 (16%)	17 (15%)	27 (23%)	
2-5 years (*n* = 304)	137 (45%)	78 (26%)	21 (7%)	68 (22%)	
> 5 years (*n* = 186)	91 (49%)	36 (19%)	20 (11%)	39 (21%)	
Radiation					.27
No (*n* = 399)	196 (49%)	83 (21%)	38 (9%)	82 (21%)	
Yes (*n* = 207)	85 (41%)	50 (24%)	20 (10%)	52 (25%)	
Chemotherapy					.64
No (*n* = 173)	81 (47%)	38 (22%)	20 (12%)	34 (20%)	
Yes (*n* = 433)	200 (46%)	95 (22%)	38 (9%)	100 (23%)	
Stem cell/marrow transplant					.35
No (*n* = 541)	255 (47%)	121 (22%)	50 (9%)	115 (21%)	
Yes (*n* = 65)	26 (40%)	12 (19%)	8 (12%)	19 (29%)	
Treatment status					.13
Receiving treatments (*n* = 193)	99 (51%)	38 (20%)	22 (11%)	34 (18%)	
Completed treatments (*n* = 413)	182 (44%)	95 (23%)	36 (9%)	100 (24%)	
Recurrence					.88
No (*n* = 524)	242 (46%)	113 (22%)	54 (10%)	118 (23%)	
Yes (*n* = 82)	39 (48%)	20 (24%)	7 (9%)	16 (20%)	
Current cancer status					.007
Disease free (*n* = 372)	156 (42%)	88 (24%)	32 (9%)	96 (26%)	
Existing disease (*n* = 234)	125 (53%)	45 (19%)	26 (11%)	38 (16%)	
Comorbidities					<.001
None (*n* = 221)	72 (33%)	56 (25%)	25 (11%)	68 (31%)	
One (*n* = 151)	61 (40%)	42 (28%)	12 (8%)	36 (24%)	
Two or more (*n* = 234)	148 (63%)	35 (15%)	21 (9%)	30 (13%)	
Exercise recommendation					.07
No (*n* = 376)	185 (49%)	86 (23%)	36 (10%)	69 (18%)	
Yes (*n* = 230)	96 (42%)	47 (20%)	22 (10%)	65 (28%)

**Table 4:** Motivations, regulations, and reflective processes of survivors meeting neither, aerobic-only, strength-only, or combined exercise guidelines

Variable	Neither (*n* = 281)	Aerobic (*n* = 133)	Strength (*n* = 58)	Combined (*n* = 134)
Motivational processes				
Attitude				
Aerobic	4.8 (1.4)	5.8 (0.8)	5.1 (1.3)	6.1 (0.7)
Strength	4.4 (1.5)	5.0 (1.2)	5.6 (1.1)	6.0 (0.7)
Injunctive norm				
Aerobic	5.5 (1.4)	6.1 (1.1)	5.7 (1.3)	6.3 (0.7)
Strength	5.2 (1.6)	5.5 (1.4)	5.7 (1.4)	6.1 (0.9)
Descriptive norm				
Aerobic	4.2 (1.8)	4.9 (1.5)	4.6 (1.8)	5.1 (1.5)
Strength	3.6 (1.8)	3.9 (1.9)	4.3 (1.8)	4.3 (1.7)
Perceived control				
Aerobic	5.1 (1.8)	6.0 (1.1)	5.5 (1.4)	6.5 (0.6)
Strength	5.1 (1.9)	5.7 (1.5)	5.8 (1.2)	6.3 (0.9)
Behavioral regulations				
Planning				
Aerobic	2.3 (1.9)	3.4 (2.2)	4.5 (1.9)	5.4 (1.5)
Strength	2.4 (1.9)	3.5 (2.3)	4.7 (2.1)	5.6 (1.6)
Reflective processes				
Obligation/Regret				
Aerobic	4.6 (3.0)	7.9 (2.1)	5.5 (3.0)	8.7 (1.7)
Strength	3.9 (3.0)	5.0 (3.0)	6.8 (2.7)	8.0 (2.3)
Regulation of alternatives				
Aerobic	5.5 (2.6)	6.0 (2.4)	5.1 (2.2)	6.8 (2.3)
Strength	4.4 (2.6)	4.2 (2.4)	5.7 (2.0)	6.2 (2.4)

Note. Standard deviations are presented in brackets. Post hoc comparisons are made between meeting the different guidelines. Attitudes, injunctive norms, descriptive norms, perceived control, and planning ranged from 1 to 7, obligation/regret ranged from 1 to 10.4, and regulation of alternatives ranged from 1 to 10

### Traditional comparisons between combined, aerobic, and strength versus neither guideline

The traditional post hoc comparisons of demographic and cancer variables from the multinomial regression are reported in Additional file 1: Table S1. Compared to HCS meeting neither guideline, those meeting the combined guideline were younger and more highly educated; HCS meeting the aerobic-only guideline were younger; and no demographic or cancer variables distinguished HCS meeting the strength-only guideline. The traditional post hoc comparisons of M-PAC based variables from the MANOVA are reported in Additional file 1: Table S2. Compared to HCS meeting neither guideline, those meeting the combined guideline had significantly more favorable aerobic- and strength-specific M-PAC profile on all measured variables. Those meeting the strength-only guideline also reported significantly greater values on all strength-specific variables versus those meeting neither guideline. HCS meeting the aerobic-only guideline had significantly greater aerobic-specific motivations, regulations, and reflective processes on all variables except for regulation over alternatives.

### Novel comparisons among combined, aerobic, and strength guidelines

Table 5 reports the novel post hoc comparisons of demographic and cancer variables between the different guidelines from the multinomial regression. HCS were more likely to meet the combined guideline over the aerobic-only guideline if they had no children living at home. They were also more likely to meet the combined guideline over both the aerobic and strength-only guidelines if they had completed university. HCS with no children living at home were more likely to meet the strength-only guideline over the aerobic-only guideline. Table 6 reports the novel post hoc comparisons of M-PAC based variables between the different guidelines from the MANOVA. HCS meeting the combined guideline reported significantly more favorable *strength-specific* scores than those meeting the aerobic guideline, and more favorable *aerobic-specific* scores than those meeting the strength guideline, on all motivations, regulations, and reflective processes. Aerobic-specific attitude, perceived control, obligation/regret, and regulation over alternatives were favored by HCS meeting the aerobic versus strength-only guideline. Strength-specific attitude, planning, obligation/regret, and regulation over alternatives were favored by HCS meeting the strength versus aerobic-only guideline. Finally, HCS meeting the combined guidelines reported significantly more favorable *strength-specific* perceived control, planning, and obligation/regret than those meeting the strength guideline, and more favorable *aerobic-specific* perceived control, planning, obligation/regret, and regulation over alternatives than those meeting the aerobic guideline.

**Table 5:** Multinomial regression comparing the demographic and cancer-specific correlates between the combined, aerobic-only, and strength-only guidelines

	Combined vs Aerobic		Combined vs Strength		Strength vs Aerobic	
Variable	OR (95% CI)	*p*	OR (95% CI)	*p*	OR (95% CI)	*p*
Age						
< 60 years vs ≥ 60 years	0.8 (0.4–1.9)	.79	1.6 (0.6–3.9)	.35	0.6 (0.2–1.5)	.25
Children living at home						
None vs. One or more	2.2 (1.2–3.9)	.006	0.7 (0.3–1.7)	.46	3.1 (1.3–7.2)	.010
Education						
University completed vs. not completed	2.0 (1.2–3.4)	.008	3.4 (1.7–6.6)	<.001	0.6 (0.3–1.1)	.11
Employment status						
Not retired vs. Retired	1.7 (0.8–3.8)	.16	1.3 (0.5–3.2)	.60	1.4 (0.6–3.4)	.50
Cancer type						
Leukemia & non-Hodgkin lymphoma vs. Hodgkin lymphoma	1.1 (0.6–1.9)	.74	0.8 (0.4–1.6)	.47	1.4 (0.7–3.0)	.33
Current cancer status						
Disease free vs. Existing disease	1.1 (0.6–1.9)	.78	1.8 (0.9–3.8)	.10	0.6 (0.3–1.2)	.15
Comorbidities						
None vs. one or more	1.4 (0.8–2.4)	.27	0.9 (0.4–1.9)	.78	1.5 (0.7–3.1)	.26

Note. *OR* odds ratio, *CI* confidence interval. All comparisons are in reference to the second listed group in each dyad

**Table 6:** Pairwise comparisons of exercise-specific motivations, regulations, and reflective processes, between combined, aerobic-only, and strength-only guidelines

Variable	Combined vs Aerobic	Combined vs Strength	Aerobic vs Strength
Motivational processes			
Attitude			
Aerobic	*p* = .07, *d* = 0.35	***p*** **< .001, d = 0.90**	***p*** **< .001,** ***d*** **= 0.63** ^a^
Strength	***p*** **< .001, d = 0.98**	*p* = .05, *d* = 0.41	***p*** **= .004,** ***d*** **= 0.50** ^b^
Injunctive norm			
Aerobic	*p* = .15, *d* = 0.24	***p*** **= .002, d = 0.57**	***p*** **= .05,** ***d*** **= 0.32** ^a^
Strength	***p*** **< .001, d = 0.53**	*p* = .07, *d* = 0.35	***p*** **= .31,** ***d*** **= 0.16** ^b^
Descriptive norm			
Aerobic	*p* = .25, *d* = 0.16	***p*** **= .042, d = 0.32**	***p*** **= .26,** ***d*** **= 0.18** ^a^
Strength	***p*** **= .05, d = 0. 24**	*p* = .89, *d* = 0.02	***p*** **= .17,** ***d*** **= 0.21** ^b^
Perceived control			
Aerobic	*p* = .010, *d* = 0.50	***p*** **< .001, d = 0.92**	***p*** **= .012,** ***d*** **= 0.44** ^a^
Strength	***p*** **< .001, d = 0.56**	*p* = .042, *d* = 0.49	***p*** **= .48,** ***d*** **= 0.13** ^b^
Behavioral regulations			
Planning			
Aerobic	*p* < .001, *d* = 1.02	***p*** **= .005, d = 0.49**	***p*** **< .001,** ***d*** **= 0.52** ^b^
Strength	***p*** **< .001, d = 1.06**	*p* = .003, *d* = 0.50	***p*** **< .001,** ***d*** **= 0.54** ^b^
Reflective processes			
Obligation/Regret			
Aerobic	*p* = .013, *d* = 0.42	***p*** **< .001, d = 1.30**	***p*** **< .001,** ***d*** **= 0.93** ^a^
Strength	***p*** **< .001, d = 1.10**	*p* = .011, *d* = 0.45	***p*** **< .001,** ***d*** **= 0.62** ^b^
Regulation of alternatives			
Aerobic	*p* = .012, *d* = 0.33	***p*** **< .001, d = 0.75**	***p*** **= .016,** ***d*** **= 0.40** ^a^
Strength	***p*** **< .001, d = 0.86**	*p* = .18, *d* = 0.23	***p*** **< .001,** ***d*** **= 0.70** ^b^

Note. ^a^= comparisons favoring the aerobic-only guideline group, ^b^= comparisons favoring the strength-only group. Hypothesized comparisons are bolded

## Discussion

The purpose of this investigation was to estimate how many HCS met the combined, aerobic-only, strength-only, and neither exercise guideline, and to examine what differentiates these four exercise groups. We previously reported in our two separate papers that 44% of HCS met the aerobic and 32% met the strength guideline [7, 8]. Our new results demonstrate that only 22% of HCS in our sample met the combined guidelines, 22% aerobic, 10% strength, and 46% neither guideline. These current results address a key limitation of our prior findings, and other studies examining aerobic and strength exercise separately, by accounting for the contamination that exists in a binary grouping scheme. For example, when examining the correlates of meeting the aerobic guideline separately, some of those categorized as meeting the aerobic guideline were in fact meeting the combined guideline, and some of those categorized as not meeting the aerobic guideline were in fact meeting the strength guideline. Thus, categorizing exercise guideline adherence into four categories avoids such confound, which also has implications for quantifying the I-B gap.

Specifically, the current investigation highlights that only about 40% of HCS who intended to meet either the aerobic, strength, or combined guideline followed-through on their intention, whereas our previous separate reports indicated that 60% of HCS successfully translated their aerobic exercise intention and 50% realized their strength exercise intention [7, 8]. Not only do we contend that the current results depict a more accurate illustration of the true I-B gap for HCS, but noting that almost no survivors (2%) met the combined guideline without an intention to do so, supports a common criticism of the intention construct: that an intention is necessary but rarely dictates behavior alone [18–20]. Furthermore, HCS who intended to meet the combined guideline, rather than just the aerobic or strength guideline, were more likely to meet at least one of these exercise guidelines. So it appears that a necessary first step towards helping survivors meet the combined guideline is to aid their formation of an intention to do both regular aerobic *and* strength exercise. Examining this data further, we see that HCS were twice as likely to fall short of their goal to meet the combined guideline because they failed to do enough strength exercise (24%) versus failing to meet the aerobic requirement (12%).

As expected, our results provided overall support for the traditional hypotheses that HCS meeting the combined, aerobic, or strength guideline would differ on key demographic, cancer, and M-PAC based variables, versus those meeting neither guideline. As commonly found in the general literature, age and education were important correlates of exercise versus no exercise [21]. Specifically, age and education status differentiated HCS meeting the combined guideline from those meeting neither, and education differentiated HCS meeting the aerobic-only guideline from those meeting neither. Interestingly, no demographic or cancer variables distinguished HCS meeting the strength guideline from those meeting neither guideline, which may suggest that M-PAC based variables may be of greater importance for driving strength exercise behavior [7]. In terms of M-PAC profile differences, compared to HCS meeting neither guideline, those meeting the combined guideline had more favorable *aerobic-* and *strength-specific* M-PAC profiles, and those meeting the strength guidelines reported an overall more favorable *strength-specific* profile. Interestingly, HCS meeting the aerobic guideline reported a similar trend for *aerobic-specific* M-PAC based variables, however, their reported level self-regulation over alternative activities did not differ significantly from those meeting neither guideline. We speculate that this may reflect a unique facet of aerobic exercise which allows individuals to multi-task while participating. So, sacrificing one’s involvement in competing activities (e.g., television watching) in order to exercise may not be required if multiple aims can be pursued simultaneously [22].

Our novel hypotheses compared HCS meeting the combined guideline, the aerobic-only guideline, and the strength-only guideline. HCS who completed university were twice as likely to meet the combined guideline over the aerobic-only guideline and three times as likely over the strength-only guideline. Thus, completing university may be associated with a greater awareness of the benefits of doing both regular aerobic and strength exercise, or may relate to better access to necessary resources (i.e., equipment, facilities) [21, 23]. Furthermore, HCS with no children living at home were significantly more likely to meet the combined guideline (two times) and strength-only guideline (three times) than the aerobic-only guideline. Exercise correlates research suggests that not having to care for dependents at home may alleviate some exercise-related time constraints [24, 25], but why this is more important for doing strength exercise over aerobic is unclear, especially when considering that the strength guideline can be satisfied in less overall time than the aerobic guideline.

As hypothesized, HCS meeting the combined guideline reported more favorable ratings on all *strength-specific* M-PAC based variables than those meeting the aerobic-only guideline, and the same was true when comparing all *aerobic-specific* variables versus those meeting the strength guideline. The most intriguing finding from our novel comparisons, however, is that HCS meeting the combined guidelines reported significantly greater *aerobic-specific* ratings of perceived control, planning, and obligation/regret than those meeting the *aerobic-only guideline*. Furthermore, this identical trend resulted for *strength-specific* ratings versus those meeting the *strength-only guideline*. Thus, these results suggest that efforts targeted towards helping HCS meet the combined guideline when already adhering to one guideline should focus on promoting both exercise modalities and not just the one in which they are deficient. For example, significant improvements in *aerobic-specific* perceived control, regulations, and reflective processes may help HCS meet the combined guideline, *even if they already meet the aerobic guideline*. Thus, we may need to reconsider the intuitive approach of only promoting the motivations, regulations, and reflective processes for the “deficient guideline,” and consider the additional need to promote (or “top-up”) the currently “performed guideline.” Altogether, these results speak to the overall benefit of using action control models (such as the M-PAC) [26], as the majority of the differentiating features between HCS meeting the combined guideline versus either singular guideline were behavioral regulations and reflective processes that are not typically captured in more traditional models (such as the TPB) [27].

Our study has important strengths and limitations. The strengths of our study include being one of the few to quantify adherence to the four categories of the exercise guidelines, the first to examine the I-B gap and M-PAC correlates in such a context, one of the few to examine the correlates of exercise in HCS, the large population-based sample of HCS, and the validated measures for social cognitive variables specific to both aerobic and strength exercise. The limitations of this study include a potential self-selection sample bias, the use of self-report measures of exercise, the cross-sectional design, and not measuring additional potentially important variables.

Our sample may have been biased due to self-selection. Though the HCS who completed the survey were not significantly different than non-respondents in age, sex, disease stage, and time since diagnosis, they likely had more favorable exercise-specific M-PAC profiles, as well as higher rates of exercise intention and participation. These biases may not only have affected our estimates of the prevalence of exercise intentions and behavior but also their associations with the correlates of meeting the exercise guidelines. Therefore, it is unclear if our findings generalize to the broader population of less motivated and active HCS. The use of self-reported measures could be influenced by recall and reporting biases which may have prompted participants to over-report their actual levels of exercise participation and motivation. Furthermore, to date, no validated or sufficiently detailed self-report measure of strength exercise exists, and we are therefore unable to comment on the quality of participants’ strength training programs. By employing a cross-sectional design, we were unable to examine the causal sequencing or hierarchy of variables in relation to participants’ exercise levels. Finally, our survey did not assess other potentially important variables such as participants’ knowledge of the exercise guidelines, their exercise history before diagnosis, and exercise habits. These variables could influence the likelihood of HCS meeting the exercise guidelines and could have been used to additionally discern whether survivors were recent exercise adopters or long-term maintainers.

## Conclusions

In conclusion, we took a novel approach to examining the correlates of exercise behavior by simultaneously analyzing the aerobic and strength exercise guidelines. Our results revealed that 22% of HCS met the combined exercise guideline, 22% aerobic-only, 10% strength-only, and 46% met neither guideline. Having no children living at home and more formal education emerged as important correlates of meeting the combined over the aerobic- or strength-only guideline. HCS meeting the combined guideline also reported more favorable ratings on all *strength-specific* M-PAC based variables than those meeting the aerobic-only guideline, and all aerobic-specific variables than those meeting the strength guideline. To help HCS meet the combined guidelines, it appears important to promote increased motivations, regulations, and reflective processes for *both* types of exercise including the exercise guideline that they are already meeting. These results may be helpful for designing health-promotion interventions aimed at helping HCS meet the aerobic and strength guidelines, thereby optimizing health outcomes.

## Additional file

Additional file 1: Table S1. Multinomial regression of demographic and cancer-specific correlates comparing combined, aerobic-only, and strength-only guidelines versus neither. **Table S2**. Pairwise comparisons of exercise-specific motivations, regulations, and reflective processes versus neither guideline. (DOCX 22 kb)
