# Supplementary material for: Correlates of meeting the combined and independent aerobic and strength exercise guidelines in hematologic cancer survivors
Source: Int J Behav Nutr Phys Act. 2017 Mar 28;14:44. doi: 10.1186/s12966-017-0498-7 (PMC5371229; doi:10.1186/s12966-017-0498-7)
Supplement: Additional file 1: Table S1. — Multinomial regression of demographic and cancer-specific correlates comparing combined, aerobic-only, and strength-only guidelines versus neither. Table S2. Pairwise comparisons of exercise-specific motivations, regulations, and reflective processes versus neither guideline. (DOCX 22 kb) [file 12966_2017_498_MOESM1_ESM.docx]

Additional file 1

Table S1. Multinomial regression of demographic and cancer-specific correlates comparing combined, aerobic-only, and strength-only guidelines versus neither.

|  | Combined vs Neither | | Aerobic vs Neither | | Strength vs Neither | |
| --- | --- | --- | --- | --- | --- | --- |
| Variable | OR (95% CI) | *p* | OR (95% CI) | *p* | OR (95% CI) | *p* |
| **Age** |  |  |  |  |  |  |
| < 60 years vs ≥ 60 years | 2.3 (1.2 – 4.4) | .010 | 2.6 (1.4 – 4.9) | .004 | 1.5 (0.7 – 3.4) | .35 |
| **Children living at home** |  |  |  |  |  |  |
| None vs. One or more | 1.5 (0.9 – 2.7) | .12 | 0.7 (0.4 – 1.2) | .17 | 2.1 (0.9 - 4.9) | .07 |
| **Education** |  |  |  |  |  |  |
| University completed vs. not completed | 2.8 (1.7 – 4.4) | <.001 | 1.4 (0.9 – 2.1) | .16 | 0.8 (0.4 – 1.5) | .46 |
| **Employment status** |  |  |  |  |  |  |
| Not retired vs. Retired | 1.4 (0.7 – 2.7) | .30 | 0.8 (0.4 – 1.5) | .49 | 1.1 (0.5 – 2.4) | .82 |
| **Cancer type** |  |  |  |  |  |  |
| Leukemia & non-Hodgkin lymphoma vs. Hodgkin lymphoma | 1.4 (0.8 – 2.3) | .22 | 1.3 (0.8 – 2.1) | .38 | 1.8 (0.9 – 3.6) | .09 |
| **Current cancer status** |  |  |  |  |  |  |
| Disease free vs. Existing disease | 1.2 (0.7 – 2.0) | .44 | 1.1 (0.7 – 1.8) | .62 | 0.7 (0.4 – 1.3) | .22 |
| **Comorbidities** |  |  |  |  |  |  |
| None vs. one or more | 1.8 (1.1 – 2.9) | .028 | 1.3 (0.8 – 2.1) | .33 | 2.0 (0.9 – 3.8) | .05 |

Note. OR = odds ratio, CI = confidence interval. All comparisons are in reference to the second listed group in each dyad.

Table S2. Pairwise comparisons of exercise-specific motivations, regulations, and reflective processes versus neither guideline

| Variable | Combined vs Neither | Aerobic vs Neither | Strength vs Neither |
| --- | --- | --- | --- |
| ***Motivational Processes*** |  |  |  |
| **Attitude** |  |  |  |
| Aerobic | ***p* < .001, *d* = 1.17** | ***p* < .001, d = 0.88** | *p* = .026, *d* = 0.22 |
| Strength | ***p* < .001, d = 1.37** | *p* < .001, *d* = 0.44 | ***p* < .001, *d* = 0.91** |
| **Injunctive norm** |  |  |  |
| Aerobic | ***p* < .001, *d* = 0.72** | ***p* < .001, d = 0.48** | *p* = .27, *d* = 0.15 |
| Strength | ***p* < .001, d = 0.69** | *p* = .05, *d* = 0.20 | ***p* = .012, *d* = 0.33** |
| **Descriptive norm** |  |  |  |
| Aerobic | ***p* < .001, *d* = 0.54** | ***p* < .001, d = 0.42** | *p* = .11, *d* = 0.22 |
| Strength | ***p* < .001, d = 0. 40** | *p* = .08, *d* = 0.16 | ***p* = .006, *d* = 0.39** |
| **Perceived Control** |  |  |  |
| Aerobic | ***p* < .001, *d* = 1.04** | ***p* < .001, d = 0.60** | *p* = .07, *d* = 0.24 |
| Strength | ***p* < .001, d = 0.81** | *p* < .001, *d* = 0.35 | ***p* = .001, *d* = 0.44** |
| ***Behavioral Regulations*** |  |  |  |
| **Planning** |  |  |  |
| Aerobic | ***p* < .001, *d* = 1.81** | ***p* < .001, d = 0.54** | *p* < .001, *d* = 1.16 |
| Strength | ***p* < .001, d = 1.82** | *p* < .001, *d* = 0.52 | ***p* < .001, *d* = 1.15** |
| ***Reflective Processes*** |  |  |  |
| **Obligation/Regret** |  |  |  |
| Aerobic | ***p* < .001, *d* = 1.68** | ***p* < .001, d = 1.27** | *p* = .021, *d* = 0.30 |
| Strength | ***p* < .001, d = 1.53** | *p* < .001, *d* = 0.37 | ***p* < .001, *d* = 1.02** |
| **Regulation of alternatives** |  |  |  |
| Aerobic | ***p* < .001, *d* = 0.53** | ***p* = .07, d = 0.20** | *p* = .19, *d* = 0.17 |
| Strength | ***p* < .001, d = 0.72** | *p* = .29, *d* = 0.08 | ***p* < .001, *d* = 0.56** |

*Note.* Hypothesized comparisons are bolded.
